# Full 3D modelling of pulse propagation enables efficient nonlinear frequency conversion with low energy laser pulses in a single-element tripler

**DOI:** 10.1038/srep42889

**Published:** 2017-02-22

**Authors:** Tomasz M. Kardaś, Michał Nejbauer, Paweł Wnuk, Bojan Resan, Czesław Radzewicz, Piotr Wasylczyk

**Affiliations:** 1 Institute of Experimental Physics, Faculty of Physics, University of Warsaw, ul. Pasteura 5, 02-093 Warsaw, Poland; 2 Institute of Physical Chemistry, Polish Academy of Sciences, ul. Kasprzaka 44/52, 01-224 Warsaw, Poland; 3 Lumentum Switzerland AG, 8952 Schlieren/Zurich, Switzerland; 4 School of Engineering, University of Applied Sciences and Arts Northwestern Switzerland, 5210 Windisch, Switzerland

## Abstract

Although new optical materials continue to open up access to more and more wavelength bands where femtosecond laser pulses can be generated, light frequency conversion techniques are still indispensable in filling the gaps on the ultrafast spectral scale. With high repetition rate, low pulse energy laser sources (oscillators) tight focusing is necessary for a robust wave mixing and the efficiency of broadband nonlinear conversion is limited by diffraction as well as spatial and temporal walk-off. Here we demonstrate a miniature third harmonic generator (tripler) with conversion efficiency exceeding 30%, producing 246 fs UV pulses via cascaded second order processes within a single laser beam focus. Designing this highly efficient and ultra compact frequency converter was made possible by full 3-dimentional modelling of propagation of tightly focused, broadband light fields in nonlinear and birefringent media.

While the near infrared band of ultrafast laser technology has been conquered by Ti:sapphire and Yb-doped sources, pulsed lasers in the near UV (200–400 nm) are still rare, albeit of growing importance in science (spectroscopy, femtochemistry, quantum optics) and technology (material processing, photolithography). With high light intensities readily available in ultrafast optics, second (SH) or third (TH) harmonics of the near infrared pulses generated in the nonlinear media are commonly employed. Since the third order processes usually are weak, the highest conversion efficiency reported for a direct TH generation scheme is 11% for 700 nm output with 25 μJ infrared input pulses^1^ and 6% for the UV (351 nm) output with 8 mJ fundamental pulses^2^. Instead of direct third harmonic generation in a single bulk nonlinear crystal, cascaded, phase-matched second order frequency conversion in two nonlinear crystals is preferred due to higher conversion efficiencies achievable^3^,^4^.

For low energy broadband pulses (i.e. femtosecond oscillators) intensities of the order of GW/cm^2^, necessary for efficient nonlinear conversion, can only be reached by tight focusing. In such conditions in addition to the temporal effects (dispersion and group velocity mismatch) the spatial effects (diffraction, spatial walk-off and angular phase matching acceptance) also play an important role. A simple one-dimensional set of coupled nonlinear equations for the three-wave mixing, fails to properly describe such configurations. In order to understand how to increase the frequency conversion efficiency, a careful management of these effects with a holistic model is required.

In the conventional arrangement^3^,^4^ (Fig. 1(a)) the second harmonic generated in the first nonlinear crystal is separated from the remaining fundamental wave and, after compensation of the temporal and spatial walk-off and polarization rotation, the two pulses are overlapped in the sum frequency generation (SFG) crystal. Overall efficiency of such setups reaches 10% in laboratory^3^,^4^ and commercial^5^,^6^,^7^ devices.

In the design described here, second harmonic generation (SHG), temporal and spatial walk-off compensation, polarization management and sum frequency generation all take place in a ‘sandwich’ of crystals (Fig. 1(b–d)). In the first nonlinear crystal, optimized for the efficient SHG, due to spatial walk-off and group velocity mismatch, the second harmonic and the remnant fundamental pulses travel different paths and experience different delays. Efficient third harmonic generation requires that they are again spatially and temporally overlapped in the second nonlinear crystal. To this end, the spatial walk-off is compensated in an YVO_4_ plate (having the walk-off angle above 6°). This solution is much more robust than the scheme based on inverted nonlinear crystals^8^, where the two crystals have to be independently tilted. The temporal walk-off is compensated with a birefringent plate – a calcite crystal in our case. As the highest conversion efficiencies in the nonlinear crystals of choice (BBO and LBO) are possible with type I phase matching for SHG and SFG processes, the polarization of the fundamental pulse is rotated by 90° with the quartz half-wave plate (being a single-wave plate for SH). In this configuration the angle tuning of the phase matching for SHG and SFG is performed with two perpendicular axes of rotation. Each of the crystals is glued into a separate aluminium holder (see Fig. 1d). Holders are then assembled together, leaving a small (around 0.1 mm) spaces between crystals.

Accurate approach to nonlinear pulse propagation with Unidirectional Pulse Propagation Equation (UPPE)^9^ resulted in highly accurate results in e.g. modelling of the supercontinuum generation^10^. Here, we followed the approach of G. Arisholm^11^, modified in line with the (UPPE) approach^9^,^12^ and thus *Hussar* was developed^13^ - an object-oriented Matlab framework optimized for small and medium scale problems. Its structure enables flexible and robust creation of 1, 2 and 3 + 1-dimensional models of broadband light fields propagation from intuitive building blocks (see Fig. 2(a)). Unlike other UPPE solving tools^14^ *Hussar* enables modelling of interaction of multiple pulses propagating in different directions, thus being capable of solving problems ranging from supercontinuum generation, to noncollinear three wave (NOPA) and four wave mixing. Uniquely, in several tests, *Hussar* delivered the results quantitatively comparable with experimental data.

The numerical modelling was employed to establish an optimal parameters of the frequency tippler, in particular the thickness of the nonlinear crystals. The thickness of the YVO_4_ crystal (SWC) is chosen to overlap the centre (approximately the maximum) of the spatial distribution of the SH pulse with the centre of the spatial distribution of the fundamental pulse in the SFG crystal. The thickness of the calcite plate (TWC) was chosen in such a way that the SH pulse overlaps with the fundamental pulse in the centre of SFG crystal. The 190 fs pulses with energy of 35 nJ, central wavelength of 1040 nm and variable focus spot diameter (assuming Gaussian temporal and spatial envelope) were used for simulations (see Supplementary Movie 1). An example of the simulation results for tripler with the LBO as the SHG and BBO as the SFG crystal are plotted in Fig. 2(b–d): Fig. 2(b) presents the calculated output power of the third harmonic, Fig. 2(c) shows the third harmonic pulse duration, and the optimum fundamental beam waist is plotted in Fig. 2(d) as a function of the SFG crystal thickness. The highest efficiencies are expected for 2.0 and 2.5 mm thick SHG crystals (black solid and dashed curves) while the SFG crystals thickness between 1.5–2.5 mm is preferred for short (~200 fs) TH pulse output as their duration is mainly determined by the group velocity mismatch between the fundamental and the TH pulse in the SFG crystal.

The effective nonlinear coefficient values *d*_*eff*_ used in the simulations were retrieved by fitting the simulation results of the *Hussar* package to the experimental data for the second harmonic generation process, using sample crystals from a given batch. The values of *d*_*eff*_ 22% and 8% lower from previously reported for BBO^15^ and LBO^16^ crystals, respectively, were found.

For the first tripler prototype, we chose to generate TH pulses with the duration no longer than the fundamental ones, i.e. 190 fs, slightly compromising the overall efficiency. A 1.5 mm SHG BBO crystal (θ = 23.2) and 2 mm BBO SFG crystal (θ = 32.1°) with 0.39 mm thick YVO_4_ (θ = 48°) and 1.46 mm calcite (θ = 90°, all from Crylight Photonics, Inc.) and a 1 mm zero-order waveplate (Eksma Optics) were used. All crystals were antireflection coated for 1040 and 520 nm, the SFG crystal is also AR coated for 347 nm. The crystals were mounted in metal holders and assembled by stacking them together. The one-inch diameter sandwich was mounted in a standard mirror mount for angle tuning of the phase matching condition around two perpendicular axes, independently for SHG and SFG.

The tripler was used for THG with 35 nJ, 190 fs pulses with the central wavelength of 1040 nm from an Yb:KYW laser oscillator (Ybix, Time Bandwidth Products). With the pulse propagation model, the optimum laser beam focusing conditions were determined and the tripler was placed in the 30 μm (2.75 mm Rayleigh range) beam waist focus generated with a f = 75 mm lens. The resulting maximum beam intensity was 6.5 GW/cm^2^, high enough for efficient frequency conversion but still below the damage threshold of the crystals.

For the maximum available input power of 2.7 W, as much as 640 mW of THG was generated (see Fig. 3a), corresponding to 24% overall conversion efficiency. Although the birefringent YVO_4_ plate used for the spatial walk-off compensation increases the overall thickness of the stack (to 5.4 mm – twice the Rayleigh range of the fundamental beam), it improves the TH conversion efficiency more than twofold. Measured M^2^ in two perpendicular planes was 1.3 and 2.36 (the former being similar to the fundamental beam with M^2^ < 1.2 and the latter corresponding to the TH spatial walk-off direction) with no measurable angular chirp, which demonstrates the high quality of the generated ultraviolet beam. The TH pulses were also characterized in the temporal and spectral domain – Fig. 3(b) and (c) – and the results are in good agreement with the theoretical predictions.

Although LBO has lower nonlinear coefficient than BBO^15^,^16^ and its phase matching conditions are sensitive to thermal effects, at the same time it has over seven times lower walk-off, which suggests that a simplified design, without the spatial walk-off compensation, could be possible, resulting in better overlap with the focal spot of the fundamental beam. In Fig. 4 we present the characteristics of the tripler with 2 mm LBO (ϕ = 13.1°, θ = 90°) for SHG, 0.62 mm calcite for delay compensation, 1 mm quartz waveplate and 2 mm BBO for SFG (the configuration marked with circles in Fig. 2). This configuration was chosen to maintain the time duration of the TH pulses below 250 fs (i.e. comparable with the fundamental pulse duration). Although, for this configuration beam sizes of around 21 μm were numerically found to be optimal, we used 30 μm waist to avoid exceeding the LBO crystal damage threshold^17^. Still 840 mW TH average power was measured, corresponding to 31% conversion efficiency, which is a threefold increase with respect to previously reported results. The TH pulse duration was 246 fs, spectral FWHM 1.9 nm and the M^2^ values were 1.24 and 2.47 (the latter corresponding to THG walk-off direction) with no angular chirp. As the crystals were kept at ambient conditions, with no temperature stabilization, for the LBO/BBO tripler we observed the thermal stabilization time of around 4 minutes, while in all designs the long term stability was limited by the stability of the laser only.

Using full 3D numerical modeling of femtosecond pulse propagation in nonlinear and birefringent media, we have developed a compact, single-element frequency tripler and demonstrated conversion efficiency as high as 31%. Although the results presented here are for a central wavelength of 1040 nm, our simulation engine can be readily used to redesign the setup for other pulsed sources e.g. at 800 or 1550 nm with different pulse durations.

## Methods

### Solving the pulse propagation equations

With *Hussar* software the UPPE is decoupled into a set of equations by introduction of (not necessarily slowly varying) envelops 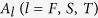 representing the fundamental, second and third harmonic waves. The introduction of envelops does not entail additional assumptions, yet it significantly reduces the amount of required computational resources and provides simple physical interpretation of the results. The propagation equations become:


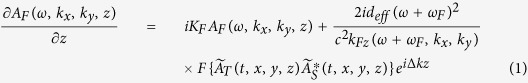



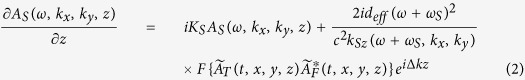



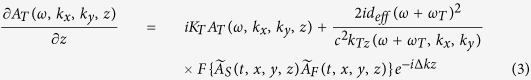


where *ω*_*l*_ are the envelopes’ central frequencies, *ω* is the frequency detuning and *k*_*x*_, *k*_*y*_ are the wavevector components^11^,^12^. The linear propagation constant 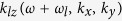 is calculated with the iterative procedure from the Sellmeier formula for each medium and polarization. The modified propagation constant: 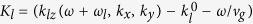, is used in the equation 1, 2, 3., where 
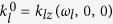
 is the reference wavenumber, *v*_*g*_ is the local time window velocity^18^ and *d*_*eff*_ is the geometry dependent effective nonlinear coefficient, 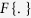 represents the 3D Fourier transform over *t, x, y*, the phase-mismatch is defined as: 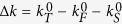. The input envelope is obtained by Fourier transform 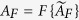 from the Gaussian beam with Gaussian temporal profile:


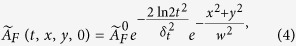


where 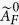 is the amplitude, *δ*_*t*_ is the pulse FWHM, and *w* is the beam waist. Eqs 1, 2, 3. are written in the Fourier space and as such form a system of ordinary differential equations for each mode of each envelope, coupled by a nonlinear polarization term. The equations were solved with the Integrating Factor^19^ Runge-Kutta 4–5 method (Dormand-Prince^20^). In each step the nonlinear polarization term is calculated by the Fourier transform, solving Eq. 2. and the inverse Fourier transform. With this spectral method the fundamental, SH and TH pulse propagation in a stack of crystals is accurately simulated. Movie 1 (frames recorded with around 30 μm steps) shows the evolution of the three pulses as they propagate through the crystal stack of the BBO/BBO tripler (the local coordinate 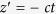 is used in the visualization).

### Tripler design optimization

For a given thicknesses of SHG and SFG crystals the thicknesses of the calcite and YVO4 crystals are selected to enable fundamental and second harmonic pulses to meet in space and time in the centre of the SFG crystal. The third harmonic pulse energy is maximized using a simplex method^21^ by varying the beam width and position of the focus with respect to the crystal stack. This procedure is iterated for different thicknesses of SHG and SFG crystals.

### Measurements

TH pulse duration was measured with the intensity cross-correlation technique involving the frequency difference generation between the TH and fundamental pulses in a 0.1 mm BBO crystal. The delay between the pulses was controlled with the Aerotech ANT95-L stage. Average powers were measured with Ophir 3A power meter and the spectra were recorded with OceanOptics USB4000 grating spectrometer with 0.5 nm resolution. M^2^ measurements were based on recording beam profiles in several planes with Basler 102f camera installed on the Aerotech PRO165LM translation stage.

## Additional Information

**How to cite this article**: Kardaś, T. M. *et al*. Full 3D modeling of pulse propagation enables efficient nonlinear frequency conversion with low energy laser pulses in a single-element tripler. *Sci. Rep.* **7**, 42889; doi: 10.1038/srep42889 (2017).

**Publisher's note:** Springer Nature remains neutral with regard to jurisdictional claims in published maps and institutional affiliations.

## Supplementary Material

Supplementary Movie 1

## Figures and Tables

**Figure 1:**
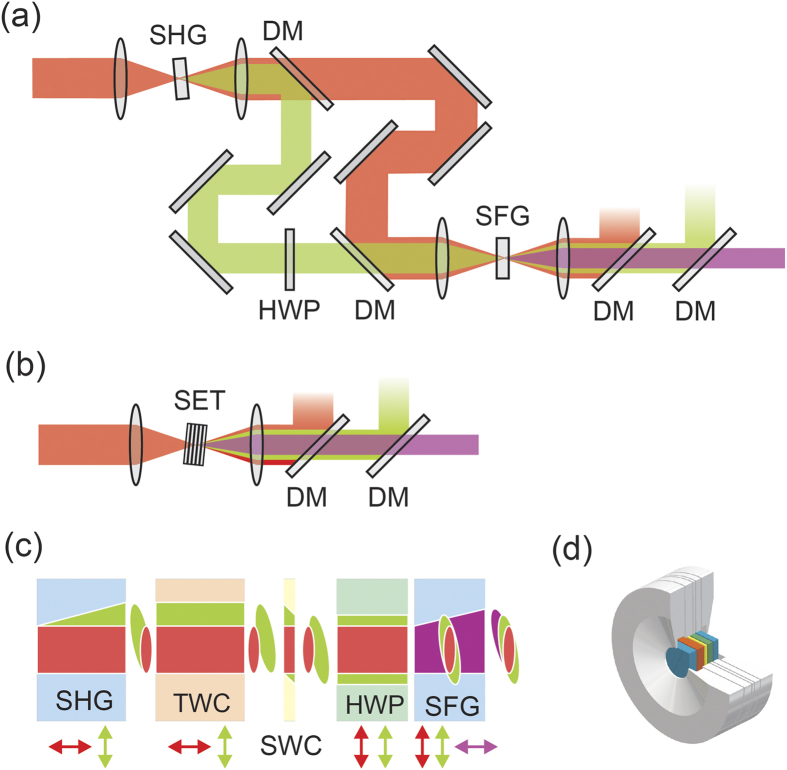
Cascaded frequency tripler. (**a**) The classical configuration, with the SH and fundamental pulses split and recombined in the SFG crystal. SHG, second harmonic (doubling) crystal; DM, dichroic mirror; HWP, half waveplate; SFG, sum frequency generation crystal. (**b**) The single-element configuration, with all the components sandwiched and placed in a single beam focus. SET, single-element tripler. (**c**), Schematic of the tripler module with the pulse paths. TWC, temporal walk-off compensation plate; SWC, spatial walk-off compensation plate. Walk-off magnitude and directions shown schematically, arrows indicate the polarization direction of the respective pulses. (**d**) Cross section of the single-element tripler with the crystals mounted in one-inch metal holder rings.

**Figure 2:**
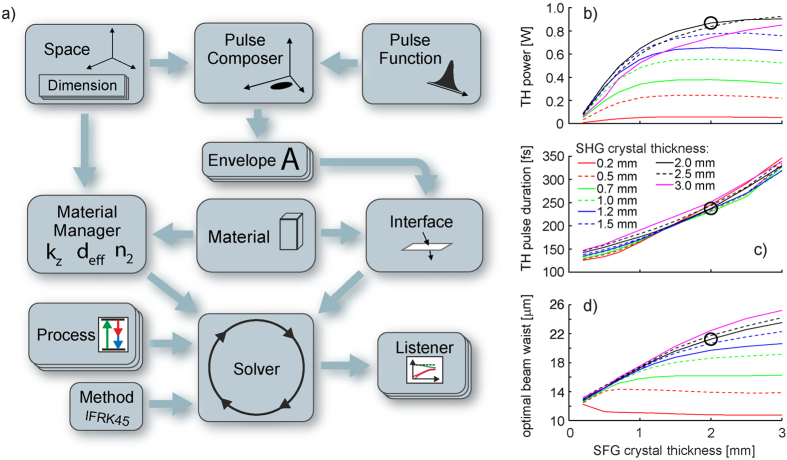
Schematic of the Hussar model generation workflow. (**a**) The model is constructed from the building blocks representing interacting pulse envelopes, material properties, the processes involved, solving methods and listeners (taking care of displaying and saving data) (**b**) Third harmonic power, (**c**) pulse duration and (**d**) the optimal beam width as a function of the SFG crystal (BBO) thickness for BBO/LBO tripler. Colors correspond to different SHG crystal thickness. Circles indicates the tripler configuration used in experiments.

**Figure 3:**
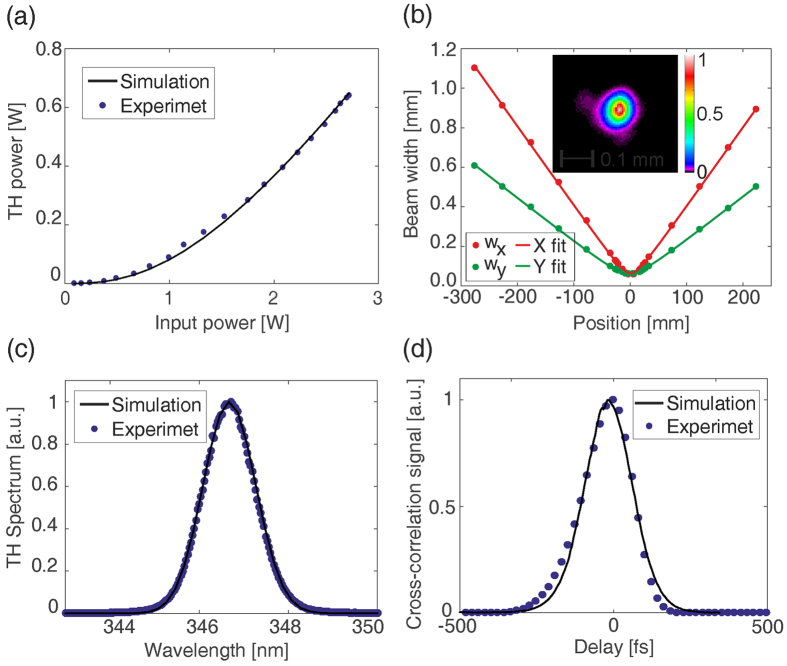
Measured and simulated TH pulse parameters for the BBO/BBO tripler. (**a**) TH average power as a function of the fundamental input average power, (**b**) the TH beam size after focusing with a f = 100 mm lens (beam profile in the focal plane shown in the inset), (**c**) the TH spectrum, (**d**) the cross-correlation of the TH and fundamental pulses. The points are measured data, the lines in (**a**,**c**) and (**d**) are the results of the simulations, the lines in (**b**) show M^2^ measurements in two planes – parallel and perpendicular to the TH polarization direction.

**Figure 4:**
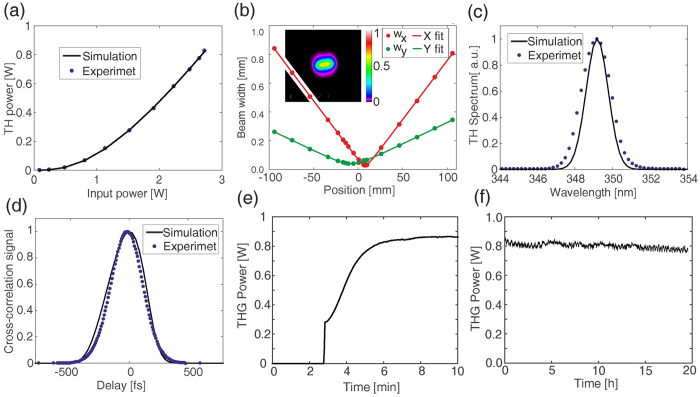
Perfomremence of the high power LBO/BBO tripler. (**a**) The TH average power as a function of the fundamental input average power, (**b**) the TH beam size after focusing with a f = 100 mm lens (beam profile in the focal plane shown in the inset), (**c**) the TH spectrum (simulation result has been shifted about 2.5 nm towards longer wavelengths to match the shape of the experimental spectrum), (**d**) the cross-correlation of the TH and fundamental pulses. (**e**) Measured TH average power as a function of time after the fundamental beam is switched on. (**f**) Long term stability of the TH average power. The points are measured data, the lines in (**a**,**c** and **d**) are the results of the simulations, the lines in (**b**) correspond to M^2^ measurements in two planes – parallel and perpendicular to the TH polarization direction.
